# Particulate Backscattering in the Global Ocean: A Comparison of Independent Assessments

**DOI:** 10.1029/2020gl090909

**Published:** 2020-12-14

**Authors:** K. M. Bisson, E. Boss, P. J. Werdell, A. Ibrahim, M. J. Behrenfeld

**Affiliations:** 1 Department of Botany and Plant Pathology, Oregon State University, Corvallis, OR, USA,; 2 School of Marine Sciences, University of Maine, Orono, ME, USA,; 3 NASA Goddard Space Flight Center, Ocean Ecology Laboratory, Greenbelt, Maryland, USA,; 4 Science Systems and Applications Inc., Lanham, MD, USA

## Abstract

How well do we know the particulate backscattering coefficient (b_bp_) in the global ocean? Satellite lidar b_bp_ has never been validated globally and few studies have compared lidar b_bp_ to b_bp_ derived from reflectances (via ocean color) or in situ observations. Here, we validate lidar b_bp_ with autonomous biogeochemical Argo floats using a decorrelation analysis to identify relevant spatiotemporal matchup scales inspired by geographical variability in the Rossby radius of deformation. We compare lidar, float, and ocean color b_bp_ at the same locations and times to assess performance. Lidar b_bp_ outperforms ocean color, with a median percent error of 18% compared to 24% in the best case and a relative bias of −11% compared to −21%, respectively. Phytoplankton carbon calculated from ocean color and lidar exhibits basin-scale differences that can reach ±50%.

## Plain Language Summary

Backscattering of light by particles is an important input for many studies concerning ecology and the carbon cycle. There are two main types of satellite sensors that measure backscattering but they have not been validated worldwide. In order to use backscattering for global questions, we need to understand how well both satellite approaches perform. Passive ocean color sensors act like wide-view cameras capturing sunlight scattered by ocean constituents, whereas active sensors use a laser system that illuminates the ocean and measures the return pulses of light within a narrow spatial range. In this study, we compare backscatter data from both satellite sensor types to matchup backscattering data collected in situ by a global network of floats. We find that backscatter data from the active and passive satellite sensors disagree, particularly at low backscattering values. Overall, the active sensor performs best when compared to field data. We applied the lidar data to reassess global phytoplankton carbon and find regional differences from conventional estimates that can reach ±50%.

## 1. Introduction

The spectral particulate backscattering coefficient (b_bp_; m^−1^; with spectral dependence hereafter implied unless noted) is central to applications of ocean optics for marine ecology and biogeochemistry. Satellite-derived b_bp_ has been used to assess particulate organic carbon (Loisel et al., 2001; Stramski et al., 1999), phytoplankton carbon (PhytoC, Behrenfeld et al., 2005; Graff et al., 2015), particle sizes (Brewin et al., 2012; Kostadinov et al., 2009; Loisel et al., 2006), and daily animal migrations (Behrenfeld et al., 2019). Satellite b_bp_ has enabled global investigations of phytoplankton physiology (via the cellular chlorophyll to carbon ratio, Behrenfeld et al., 2005), improvements in ecological models where particle size is needed (e.g., Bisson et al., 2020), advanced mechanistic net primary production algorithms (Silsbe et al., 2016; Westberry et al., 2008), determinations of carbon export (Siegel et al., 2014), and global analyses of marine biogeochemical change (e.g., Behrenfeld et al., 2006).

There are currently three ways to measure b_bp_ globally: (1) autonomous profiling floats (Bittig et al., 2019), (2) passive (or “ocean color”) satellite remote sensing, and (3) active satellite remote sensing (light detection and ranging, lidar). Recent progress in using b_bp_ derived from satellite lidar measurements (hereafter “lidar b_bp_”) to study ocean biology (Behrenfeld et al., 2013, 2017, 2019; Lu et al., 2016, 2020) has established lidar as a prominent tool, such that we are now entering a “satellite lidar era in oceanography” (Hostetler et al., 2018). There is little doubt that lidar b_bp_ observations will continue to advance our understanding of ocean processes because lidar b_bp_ can observe polar ecosystems in the absence of sunlight and at low sun angles, potentially provide constraints on inversion algorithms for passive remote sensing approaches, and contribute another independent measurement of ecosystem stocks.

For decades, it was not possible to assess passive satellite performance of b_bp_ retrievals on global scales because there were few in situ observations. For example, the NASA Moderate Resolution Imaging Spectroradiometer (MODIS) onboard Aqua was launched in 2002, but its most spatially extensive b_bp_ performance assessment was not realized until 2019 (K. M. Bisson et al., 2019), when a global network of Argo floats equipped with backscattering sensors was used. Here, we conduct a similar analysis of lidar b_bp_.

Identifying in situ matchup observations with MODIS is straightforward relative to lidar because passive ocean color satellite instruments produce wide swaths of data, often stretching 2,000 km in the cross-track direction. In contrast, defining matchups for lidar and in situ observations is challenging because a single lidar pulse, like an in situ measurement, gives a snapshot in time for pinpricks in space. On regional scales, MODIS and lidar have been compared before in polar regions and in the North Atlantic (Behrenfeld et al., 2017; Lacour et al., 2020). Satellite lidar has not been validated globally. In this study, we ask, “Are ocean color and lidar b_bp_ retrievals consistent on global scales?”

Satellite b_bp_ (*λ*) needs to be contextualized with known biases and assessed errors because the fidelity of past and future modeling efforts relies on the accuracy of b_bp_ as an input product. Here, we introduce a method to globally validate lidar backscattering from the Cloud-Aerosol Lidar with Orthogonal Polarization (CALIOP) instrument aboard the NASA Cloud-Aerosol Lidar and Infrared Pathfinder Satellite Observations (CALIPSO) satellite, and we compare CALIOP b_bp_, MODIS b_bp_, and Argo b_bp_ with the goal of quantifying satellite b_bp_ performance and bias.

## 2. Materials and Methods

### 2.1. Argo b_bp_

Vertical profiles of b_bp_ (700 nm, m^−1^) were downloaded from the Argo Data Assembly Center (ftp://ftp.ifremer.fr/ifremer/argo/dac/ on May 20, 2020) and processed as in K. M. Bisson et al. (2019). Float b_bp_ profiles were despiked with a three-point moving median and outliers in log-space were removed (given by those b_bp_ values outside the bounds of 1.5 times the interquartile range). After outliers were removed, there were 37,337 data points at independent locations. To make the float profiles comparable to remote sensing products (where CALIOP data represent a fixed 22.5 m layer, and MODIS data are exponentially weighted toward the surface), the mean b_bp_ value is reported within the calculated mixed layer depth (MLD, given by the depth where density is greater than 0.03 kg m^−3^ relative to the density at 10 m). The median MLD is 18 m for the global Argo data set, with an interquartile range of 3.9 m. Choosing the first light attenuation layer rather than the MLD did not significantly change the values of Argo b_bp_.

### 2.2. Retrieving Ocean Color b_bp_

The retrieval of ocean color b_bp_(*λ*) is an ill-posed inverse problem that requires spectral remote sensing reflectances (R_rs_(*λ*); sr^−1^) as input and is constrained with a set of assumptions about the absorbing and backscattering constituents in the ocean. Our analysis is focused on the MODIS instrument onboard Aqua and the generalized inherent optical properties algorithm in its default configuration (GIOP-DC, Werdell et al., 2013) because MODIS outperformed the other contemporary global ocean color satellites such as Visible Infrared Imaging Radiometer Suite (VIIRS) and Ocean and Land Color Instrument (OLCI) in K. M. Bisson et al. (2019). Likewise, the GIOP-DC outperformed the other inversion algorithms such as Quasi Analytical Algorithm (Lee et al., 2002) and Garver–Siegel–Maritorena algorithm (Maritorena et al., 2002) when confronted with Argo float b_bp_ in K. M. Bisson et al. (2019).

We obtained MODIS Level-3 9-km remote sensing reflectance data (R_rs,_ *λ* = 412, 443, 488, 531, 547, and 667 nm) to generate global b_bp_ maps using the GIOP-DC algorithm, as well as MODIS Level-2 1-km R_rs_ (same wavelengths, all from the NASA Ocean Biology Processing Group, https://oceancolor.gsfc.nasa.gov) to generate coincident matchups with Argo b_bp_ according to the Bailey and Werdell (2006) quality control criteria. We identified satellite matchups that occur within a ±3-h window in a 5 × 5 satellite pixel box, and also within a ±24-h window in a 9 × 9 pixel box centered on the float observation (where the larger box accounts for assumed advection). All R_rs_ (*λ*) data were corrected to remove Raman scattering through the empirical algorithm of Lee et al. (2013).

GIOP is a flexible inversion algorithm that allows different formulations within the framework to be modified (full details in Supplementary Material Text S1). We ran the GIOP-DC on MODIS R_rs_ observations and report our derived b_bp_ at 532 nm so that MODIS and CALIOP b_bp_ are compared at the same wavelength. Finally, because MODIS b_bp_ is a function of eigenvector choices for b_bp_(*λ*), phytoplankton absorption (a_ph_(*λ*); m^−1^), and nonalgal particle plus colored dissolved organic matter absorption (a_cdm_(*λ*); m^−1^), we performed a sensitivity analysis to quantify MODIS b_bp_ performance depending on which specific assumptions are used (see Supplementary Material Text S2, Table S1).

### 2.3. Satellite Lidar Retrievals of b_bp_

The CALIPSO satellite was launched in 2006 with the primary goal of observing the vertical distribution of clouds and aerosols. Like MODIS, CALIPSO flies in the A-train constellation and has a 16-day repeat cycle (Winker et al., 2009). CALIPSO’s main instrument is CALIOP, which is a nadir-pointing lidar with two measurement wavelengths, 532 nm and 1,064 nm, and has a footprint diameter at the ocean surface of ~100 m. CALIOP measures the copolarized and cross-polarized channel component of column integrated backscatter. Although CALIOP was not intended for ocean research, its polarization properties have been used to characterize b_bp_ at 532 nm for the first vertical 22.5-m bin in the ocean (Behrenfeld et al., 2013). Since 2013, there have been refinements to the lidar b_bp_ algorithm. In this analysis, we use the daytime lidar product published in Behrenfeld et al. (2019), which is freely available online (data access details are in the acknowledgments, and data processing details are in Supplementary Material Text S3). We made one key modification to the Behrenfeld et al. (2019) CALIOP b_bp_ product. The Behrenfeld et al. (2019) study used a processing factor of 0.16 for the ratio of b(*π*) to b_bp_. More recent work (Lacour et al., 2020; Lu et al., 2020) used a constant value of 0.32.

In our study we choose a beta(*π*)/b_bp_ value of 0.32. Because the Behrenfeld et al. (2019) CALIOP data were processed using a value of 0.16, we multiply the retrieved b_bp_ product by 0.5. Using this factor, the global b_bp_ frequency distributions between CALIOP and MODIS are similar (Figure S1). Given this, we focus our efforts on point by point comparisons of spatiotemporal matchups common to CALIOP, MODIS, and Argo observations. Argo b_bp_ is used from 2015 to present, and the CALIOP b_bp_ product used in this study spans 2006–2017, so we restrict our analysis to 2015–2017.

### 2.4. Identifying Matchups Across CALIOP, MODIS, and Argo b_bp_

**Figure 1.**
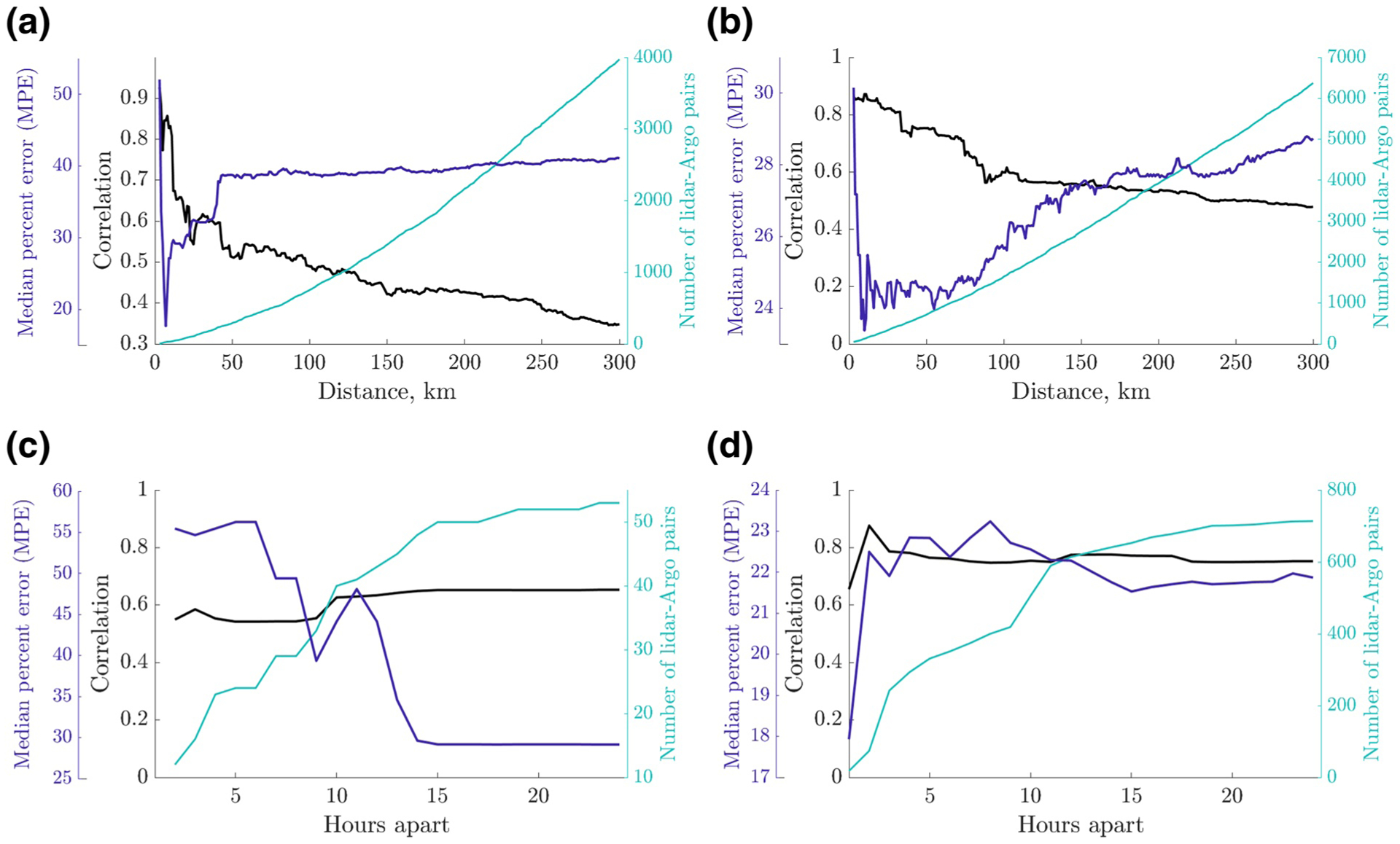
Correlation (black) and median percent error (purple) distances (a, b) and times (c, d) between CALIOP and Argo b_bp_ for areas with mean ocean temperature < and >15°C (left and right panels, respectively). Cyan lines correspond to the number of observations at a given distance or time. (a, b) Argo and CALIOP are ±24 h apart. (c) Argo and CALIOP are 15 km apart. (d) Argo and CALIOP are 50 km apart. CALIOP, Cloud-Aerosol Lidar with Orthogonal Polarization.

Observations from CALIOP and Argo are single points separated by distance and time, so we could not use a method that relies on their intersection for comparison. Instead, we adopted a decorrelation approach to quantify near-coincident space (Figures 1(a) and 1(b), black and purple lines) and time windows (Figures 1(c) and 1(d), black and purple lines) that yield sufficient matchups (cyan lines) for analysis. Rather than group all data together, we chose to subset regions by annually averaged sea surface temperature (SST), where an SST of 15°C was used to distinguish warmer waters that are permanently stratified within the euphotic layer from cooler, high latitude waters with deeper active mixing (after Behrenfeld et al., 2006). Regions with annual SST < 15°C (Figures 1(a) and 1(c)) and >15°C (Figures 1(b) and 1(d)) represent different physical environments because the first baroclinic Rossby radius of deformation (defining the length scale of baroclinic variability) is dependent on the Coriolis parameter (and therefore on latitude, Chelton et al., 1998). Higher latitudes are expected to exhibit shorter decorrelation length scales of physical variability, which are expected to influence the decorrelation in b_bp_.

We calculated distances (in km) between Argo and CALIOP using the haversine formula. Pearson’s correlation (r) is used to quantify similarity between CALIOP and Argo b_bp_ on log-10 transformed data. We defined coincidence with MODIS following Bailey and Werdell (2006). Backscattering spectral slopes (*γ*) calculated as part of the GIOP-DC inversion were applied to the Argo b_bp_ at 700 nm to derive Argo b_bp_ at 532 nm so that all b_bp_ are comparable at the same wavelength (see Equation 4 in Supplementary Material Text S1). We calculated the median percent error (MPE, the median of 100% × |satellite b_bp_/Argo b_bp_ – 1|) and relative bias (the median of 100% × [satellite b_bp_ − Argo b_bp_]/Argo b_bp_) to compare MODIS, CALIOP, and Argo b_bp_ (all at 532 nm; m^−1^, and data are not log-transformed prior to these calculations because the data are normalized by Argo b_bp_).

We used the shapes of decorrelation for r and MPE to find cutoff distance values where the slope of MPE increases and the slope of the correlation decreases (Figure 1). The intent of this approach was to maximize the number of paired observations while maintaining a high correlation and low MPE. Based on this analysis, we chose 15 km radius for matchups in regions with annual SST < 15°C and 50 km for regions with annual SST > 15°C (Figure 1). In all cases, the correlation is similar across all hours (up to 24). With these matchup criteria, we take a subset of the Argo observations common to both CALIOP and MODIS b_bp_ (within a ±3-h window, *n* = 93 as well as ±24-h window, *n* = 261) so that all three sensor types can be compared. One alternative approach to the paired matchup method as outlined here is to look at general correspondence between distributions of CALIOP and Argo b_bp_ in particular regions, as is done in Lacour et al. (2020) in the North Atlantic. Differences between Lacour et al. (2020) and the current study are discussed further in Supplementary Material Text S4.

## 3. Results

**Figure 2.**
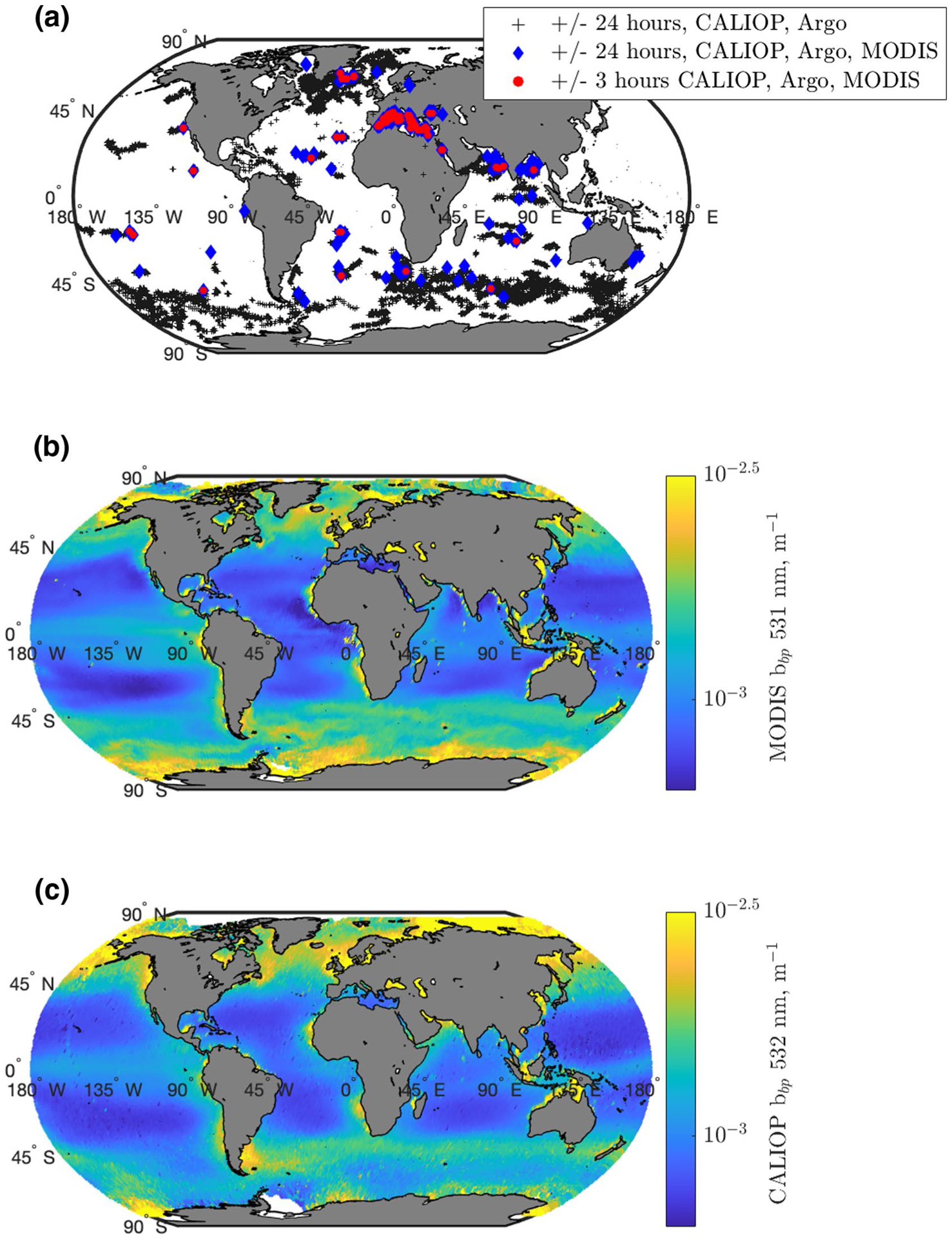
(a). Map of locations with coinciding Argo floats and either CALIOP observations (black plus) or CALIOP and MODIS L2 imagery within 24 h (blue, *n* = 261) or 3 h (red, *n* = 93). (b). Annually averaged MODIS b_bp_ (531 nm, m^−1^), constructed from L3 9 km files binned to a 1-degree grid. (c). Annually averaged CALIOP b_bp_ (532 nm, m^−1^), constructed from CALIOP observations binned to a 1-degree grid. CALIOP, Cloud-Aerosol Lidar with Orthogonal Polarization; MODIS, Moderate Resolution Imaging Spectroradiometer.

The spatial distribution of matchup Argo b_bp_ observations common to both CALIOP and MODIS exhibits good global coverage with representation in the Southern Ocean, Arctic Ocean, South Pacific Gyre, Atlantic basin, and Indian Ocean (Figure 2(a)). Global annually averaged maps of MODIS and CALIOP b_bp_ reveal similar patterns (Figures 2(b) and 2(c)), with elevated b_bp_ in coastal and/or upwelling regions and lower b_bp_ in the oligotrophic gyres.

**Figure 3.**
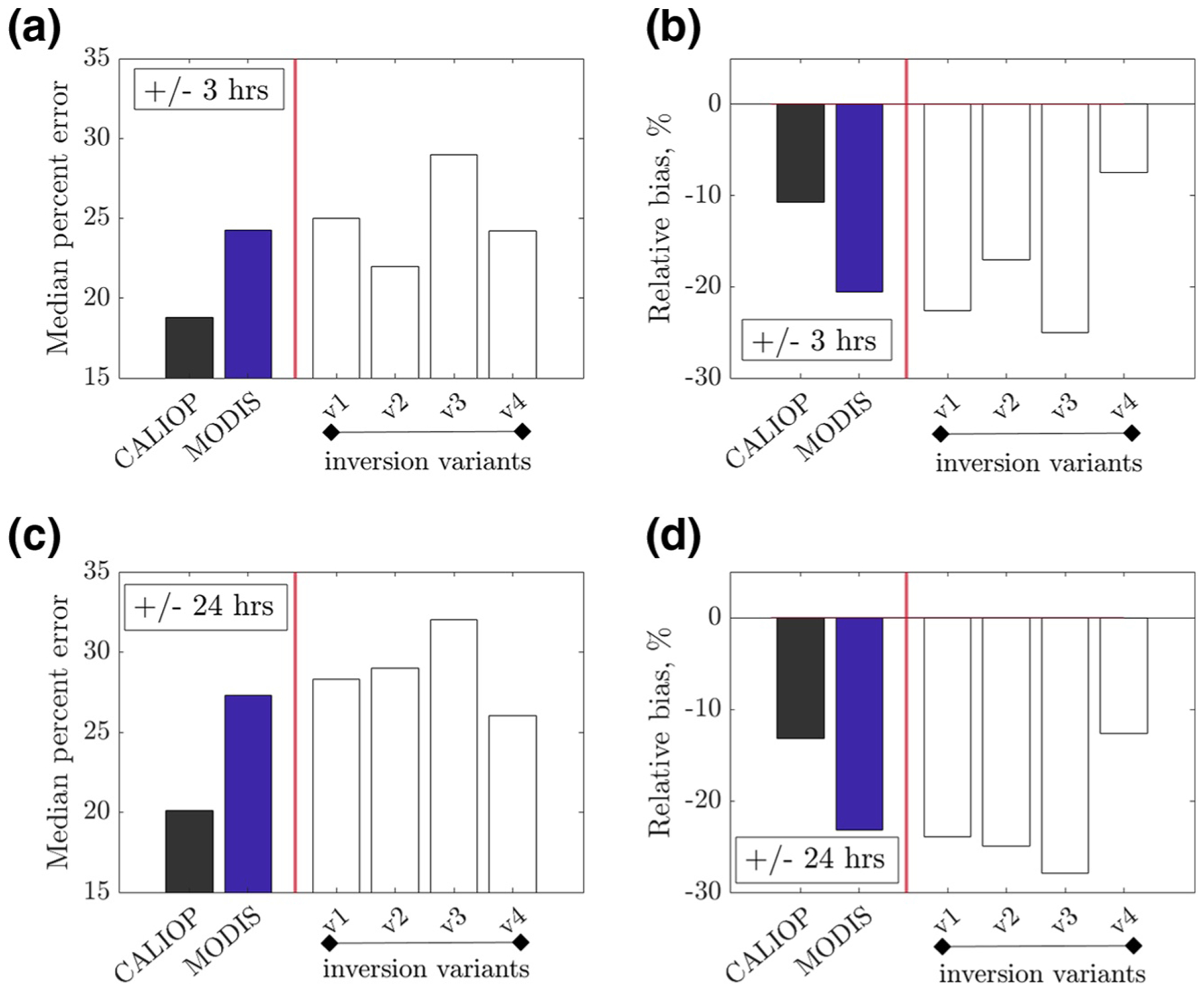
Comparison of CALIOP performance metrics and those for variants of MODIS inversions. V1 changes the b_bp_ slope used, V2 changes the a_cdm_ shape, V3 changes the assumed a_ph_ shape, and V4 is the GIOP result using R_rs_ data that were not corrected for Raman scattering. (a), (b) +/−3 h matchup data. (c), (d) +/−24 h matchup data. (a), (c) median percent error. (b), (d) relative bias (%). In all panels, black bar is CALIOP and purple bar is for MODIS using the optimum (default) configuration of the GIOP algorithm. Red vertical line separates results for this GIOP configuration from other inversion variants (v1, v2, v3, and v4—see Supplementary Text S2 and Table S1 for description of variants). CALIOP, Cloud-Aerosol Lidar with Orthogonal Polarization; MODIS, Moderate Resolution Imaging Spectroradiometer; GIOP, generalized inherent optical properties.

Evaluation of equivalent matchup data between Argo observations of b_bp_ and retrievals from CALIOP and the optimum parameterization of MODIS GIOP-DC reveals a superior performance of CALIOP (black bars vs. purple bars in Figure 3), having lower MPE (Figures 3(a) and 3(c)) and relative bias (Figures 3(b) and 3(d)). This improved performance is observed both for the 3-h matchup data (where CALIOP has 18% MPE vs. 24% MPE for MODIS [Figures 3(a), S1(a), and S1(c)] and CALIOP has a lower relative bias [−11%] compared to MODIS [−21%] [Figure 3(b))] and the 24-h matchup data (Figures 3(c), 3(d), S2(b), and S2(d)). CALIOP also exhibits superior performance in b_bp_ retrievals compared to four alternative ocean color inversion (GIOP) parameterizations (MPE and relative bias bars to the right of the vertical red line in Figure 3).

We find clear inconsistencies between CALIOP and MODIS b_bp_ (Figures S2(c) and S2(g)). For b_bp_ values around 0.001 m^−1^, MODIS exhibits a higher dynamic range of b_bp_ compared to CALIOP, spanning nearly an order of magnitude in the ±24-h case (Figure S2(g)).

In a qualitative sense, MPE and bias are better for both sensors in the ±3-h window compared to the ±24-h window, and both MODIS and CALIOP underestimate Argo b_bp_ in general (Figures 3(b), 3(d), S2, and S3).

**Figure 4.**
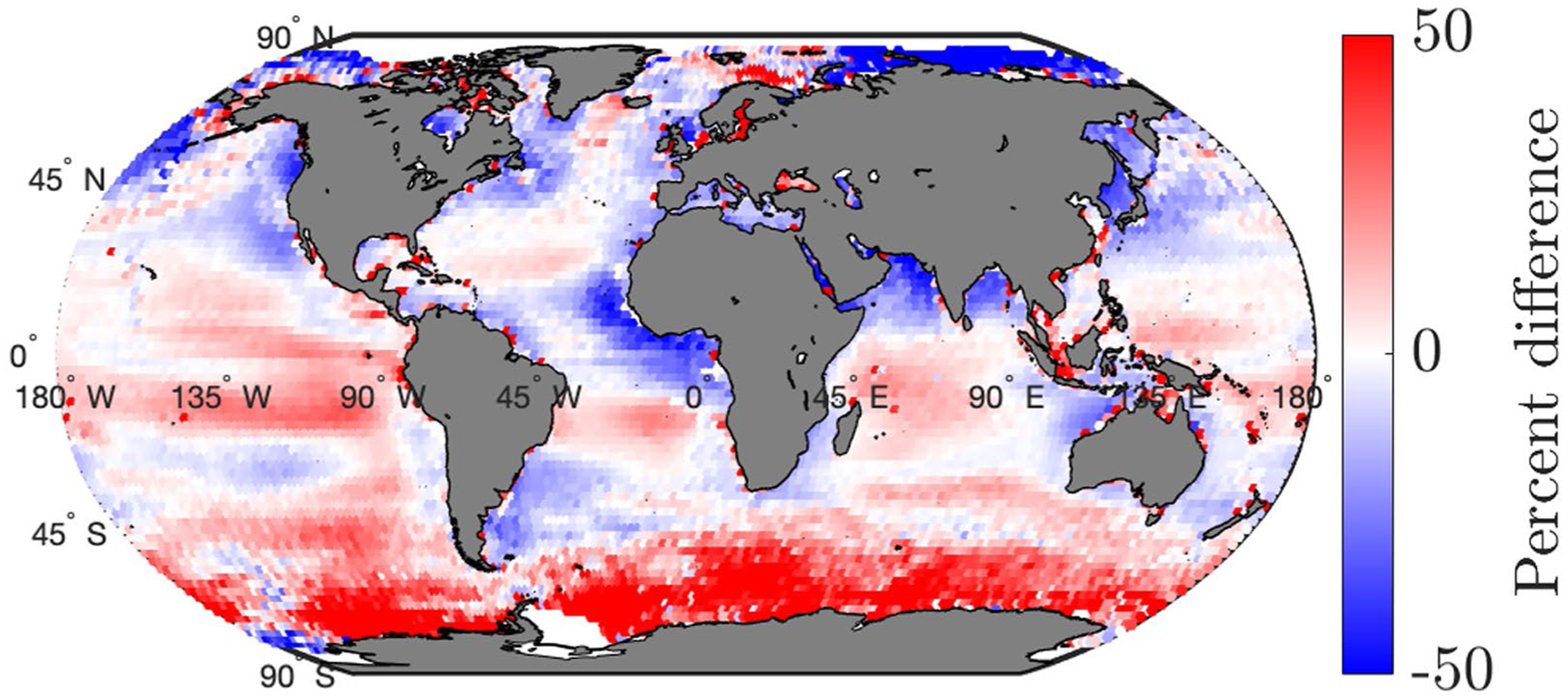
Annually averaged relative percent difference in PhytoC between MODIS and CALIOP, relative to CALIOP (i.e., 100 × [PhytoC_M_ – PhytoC_C_]/PhytoC_C_). MODIS, Moderate Resolution Imaging Spectroradiometer; CALIOP, Cloud-Aerosol Lidar with Orthogonal Polarization.

As an illustration of the ecological and biogeochemical significance of CALIOP and MODIS b_bp_ differences, we converted these data into estimates of PhytoC concentrations using the linear relationship reported by Graff et al. (2015). While annual global average PhytoC estimates from MODIS (PhytoC_M_) and CALIOP (PhytoC_C_) are similar (17 mg C m^−3^ and 18 mg C m^−3^, respectively), notable regional differences are observed (Figure 4). For example, PhytoC_C_ is ~20% higher than PhytoC_M_ in the South Pacific gyre and temperate regions of the North Pacific and South Atlantic. In contrast, PhytoC_M_ exceeds PhytoC_C_ by ~20% in the Equatorial Pacific and the central gyres of the South Atlantic and Indian Oceans. The largest differences between retrievals are found in the North Indian Ocean, the equatorial Atlantic west of Africa, and the Artic/Subarctic, where PhytoC_C_ may exceed PhytoC_M_ by up to 50%, and in the Southern Ocean where PhytoC_M_ exceeds PhytoC_C_ by 50%.

## 4. Discussion

The improved performance of lidar b_bp_ retrievals relative to ocean color reported here is a somewhat unexpected finding because spatial coverage of lidar data is so restricted compared to ocean color data. In other words, the average spatiotemporal coincidence between Argo b_bp_ data and lidar is far broader than that for wide-swath, 2-day repeat cycle ocean color measurements, suggesting (a priori) that ocean color b_bp_ should yield better performance when compared to float data, at least in spatiotemporal heterogenous waters. Instead, the relatively low MPE (18%) and relative bias (−11%) for the CALIOP data is a clear improvement over all contemporary ocean color satellites, even when considering their highest performing b_bp_ algorithm (MODIS-24% MPE, bias = −21%, this study, VIIRS-31% MPE, bias = −11% [K. M. Bisson et al., 2019], and OLCI-45% MPE, bias = 2% [K. M. Bisson et al., 2019], with biases recalculated according to our definition here, Figure S1). Although the study of K. M. Bisson et al. (2019) featured more matchups between Argo and ocean color b_bp_, we note that lidar MPEs are below 25% even for ±24-h matchups at distances >50 km in the SST > 15°C case, which represents ~75% of the ocean by area. We also note that MODIS performance degrades with distance and time (as expected), with an MPE of 27% in the 9 × 9 pixel box, ±24-h matchup case. While the performance of MODIS b_bp_ is indeed affected by choices within the GIOP inversion, no particular configuration can produce the performance metrics of CALIOP b_bp_. If more exact spatial matchups were possible between lidar and Argo b_bp_ data, we would expect the enhanced performance of lidar compared to ocean color to be even more pronounced.

Although we have found good agreement between CALIOP and Argo b_bp_, CALIOP b_bp_ is imperfect, particularly at low Argo b_bp_ values (Figure S4). Future lidar products may especially benefit by optimizing b_bp_ to float values (as is done currently with the MODIS SST algorithm, Kilpatrick et al., 2015), especially because there are sufficient (~750) matchups between CALIOP and Argo observations. CALIOP b_bp_ is also sensitive to the scattering phase function used (which might vary regionally/seasonally) and data are only available along the orbit track as opposed to the large ocean color swaths.

There are necessarily limitations of ocean color b_bp_. Lidar is a more direct measurement of b_bp_ compared to MODIS, as the latter retrieval uses the remote sensing reflectances, R_rs_(*λ*), with assumptions about the absorption and backscattering spectral shapes of the ocean components and specific relationship between R_rs_(*λ*) and inherent optical properties. R_rs_(*λ*) is retrieved following atmospheric correction, which removes radiometric effects from ocean surface glint and white-caps, as well as molecular and aerosol absorption and scattering. The chemical composition and size distribution of aerosols are assumed (Gordon, 1997; Gordon & Wang, 1994) and inferring the aerosol signal from satellite observations can be challenging since the atmospheric signal contribution is typically 90% at 440 nm at the top of the atmosphere while the residual signal is from the ocean. Even worse, the contribution of the ocean signal quickly decreases at longer wavelengths (i.e., >500 nm), making it more challenging to accurately estimate R_rs_(*λ*). While useful for sensor-to-sensor comparisons, the bidirectional reflectance distribution function correction, as part of the atmospheric correction, can impart additional uncertainty in R_rs_(*λ*) retrievals, as it cannot ubiquitously represent all conditions at all times (Mobley et al., 2016). Small uncertainties in the aerosol correction lead to large uncertainties in R_rs_ at green and red bands due to two inherent limitations: (1) the ocean signal is small relative to the aerosol signal and (2) the dynamic range of R_rs_ in the green and red wavelengths is small compared to the more dynamic aerosol signal. A future assessment is required to quantify the impacts of atmospheric correction on b_bp_ retrievals. Despite the issues outlined above, we find a good overall correspondence between ocean color and Argo b_bp._

CALIOP, Argo, and MODIS observe b_bp_ in different areas of the volume scattering function. CALIOP is nadir viewing (scattering angle of 180°), typical backscattering sensors used on Argo float have a nominal scattering angle of 142° (but some have 124° and 149°, Poteau et al., 2017), while MODIS has viewing angles relative to nadir spanning ±49.5° (corresponding to scattering angles between 131° and 180°; https://aqua.nasa.gov/modis). As R_rs_ is known to be influenced by viewing angle and particle phase function (with variations up to 65% in some cases, Xiong et al., 2017), the viewing angle differences between sensors are a potential source of error for the retrieved b_bp_ products. Another source of discrepancy between sensors is the water column depth used to generate b_bp_ observations. MODIS, CALIOP, and Argo consider slightly different depths of the water column, which may be potentially important for instances when the sensor depth exceeds the mixing depth. A further source of error arises from the different sensor wavelengths used in this study. Given that ocean color b_bp_ performance is affected by assumed backscattering and absorption shapes, it would be a meaningful improvement for future floats to be equipped with a backscattering sensor including green wavelengths. Having Argo b_bp_ observations in the green would eliminate the influence of the backscattering spectral power-law-fit slope assumption and also the influence of absorption assumptions because the green bands are minimally influenced by phytoplankton and water absorption.

In this study, we validated global lidar b_bp_ and compared it to the best case ocean color sensor and algorithm pairing. Regional differences in derived PhytoC between CALIOP and MODIS quantify the consequences of b_bp_ product choice. PhytoC is essential for calculating phytoplankton physiology through the chlorophyll:PhytoC ratio and it is a central term in state-of-the-art NPP algorithms (i.e., Silsbe et al., 2016; Westberry et al., 2008) and carbon export models (where differences in data products have wide effects on model outcomes, K. M. Bisson et al., 2018). Although there are clear spatial differences between CALIOP and MODIS b_bp_, we choose not to focus on regional differences within our analysis because there are too few observations (93 globally, with only 5 observations poleward of 50 degrees) to make rigorous statements about performance on regional scales.

Because CALIOP has limited spatial coverage compared to MODIS, an optimal approach may come from generating products that combine data from the two sensors. Continued efforts are needed to improve CALIOP lidar retrievals in low b_bp_ areas. Nevertheless, the CALIOP record provides a less uncertain and an independent global data set of b_bp_ that presents an opportunity for evaluating and improving satellite ocean color retrievals of this fundamental optical property related to plankton ecosystem structure and biogeochemistry.

## Supplementary Material

supp

## Data Availability

All ocean color data can be downloaded at https://oceancolor.gsfc.nasa.gov. All lidar data can be downloaded at http://orca.science.oregonstate.edu/lidar_grl_2020.php. Argo data were collected and made freely available by the International Argo Program and the national programs that contribute to it (http://doi.org/10.17882/42182). The Argo Program is part of the Global Ocean Observing System.

## Key Points:

- Spatiotemporal correlation scales are quantified between global lidar and in situ observations
- Satellite lidar has lower error and bias compared to ocean color observations of particulate backscattering
- Phytoplankton carbon values determined from global lidar and ocean color differ within basins by as much as 50%
