# Supplementary material for: Particulate Backscattering in the Global Ocean: A Comparison of Independent Assessments
Source: Geophys Res Lett. Author manuscript; Available in PMC 2021 Sep 15. (PMC8442828; doi:10.1029/2020gl090909)
Supplement: supp [file NIHMS1736996-supplement-supp.docx]

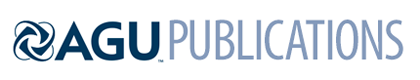


*Geophysical Research Letters*

Supporting Information for

**Particulate backscattering in the global ocean: A comparison of independent assessments**

K. M. Bisson^1^, E. Boss^2^, P. J. Werdell^3^, A. Ibrahim^3,4^ and M. J. Behrenfeld^1^

^1^Department of Botany and Plant Pathology, Oregon State University, Corvallis, Oregon, 97331, USA.

^2^School of Marine Sciences, University of Maine, Orono, Maine, 04469, USA.

^3^Ocean Ecology Laboratory, NASA Goddard Space Flight Center. Greenbelt, Maryland, 20771, USA.

^4^Science Systems and Applications Inc., Lanham, Maryland, 20706, USA.

**Contents of this file**

Text S1 to S4

Figure S1 to S4

Table S1

**Introduction**

This file contains supporting information about the ocean color b_bp_ processing. We provide full details about the inversion algorithm (Text S1), and we include a sensitivity analysis to quantify how MODIS b_bp_ values change depending on b_bp_ model assumptions used (Text S2, Table S1). We also provide details on CALIOP data processing (Text S3) and compare our study with a regional analysis of CALIOP, MODIS, and Argo (Text S4). We also provide scatter and Bland-Altman plots for the Argo, CALIOP, MODIS comparisons (Figures S3, S4).

Text S1. Description of R_rs_ inversion algorithm

The GIOP inversion requires R_rs_(λ) as input and generates b_bp_(λ). First, R_rs_ is converted to its subsurface values, r_rs_(λ) (Lee et al., 2002), via

$\text{r}_{\text{rs}}\left( \text{λ} \right)\text{=}\frac{\text{R}_{\text{rs}}\text{(}\text{λ}\text{)}}{\text{0.52+1.7}\text{R}_{\text{rs}}\text{(}\text{λ}\text{)}}$. [1]

The relationship between r_rs_ and b_bp_ is given by the ratio of total backscattering (b_b_) to the sum of absorption (a; m^-1^) plus b_b_, or:

$\text{r}_{\text{rs}}\left( \text{λ} \right)\text{= }\sum_{\text{ι}\text{=1}}^{\text{2}} \text{G}_{\text{i}}\text{*[}{\frac{\text{b}_{\text{b}}\text{(}\text{λ}\text{)}}{\text{a}\left( \text{λ} \right)\text{+}\text{b}_{\text{b}}\text{(}\text{λ}\text{) }}\text{]}}^{\text{i}}$ , [2]

where $G_{1}$ = 0.0949 and $G_{2}=$ 0.0794 (Gordon et al. 1988). Total absorption is given by the sum of seawater absorption (a_w_(λ); m^-1^), absorption from colored dissolved organic matter (CDOM) and non-algal particles (a_cdm_(λ); m^-1^), and phytoplankton absorption (a_ph_(λ); m^-1^), or:

$\text{a}\left( \text{λ} \right)\text{= }\text{a}_{\text{w}}\left( \text{λ} \right)\text{+}\text{M}_{\text{cdm}}\text{e}^{\text{-S}_{\text{cdm}}\lambda}\text{+}\text{M}_{\text{ph}}\text{a*}_{\text{ph}}^{\text{ }}\left( \text{λ} \right)$*,*  [3]

where a_cdm_ and a_ph_ are expressed as the product of an amplitude (M_x_) and a spectral shape function. In the default GIOP configuration, the spectral shape function for a_cdm_ is expressed with S_cdm_ = 0.018 nm^-1^ and the spectral shape function for a_ph_ is assigned using satellite-retrieved chlorophyll-a (mg m^-3^) following Bricaud et al. (1998). Total b_b_ is given by the sum of backscattering of seawater (b_bw_) and particles (b_bp_), or:

$\text{b}_{\text{b}}\left( \text{λ} \right)\text{=} \text{b}_{\text{bw}}\left( \text{λ} \right)\text{+}\text{M}_{\text{bp}}\lambda^{\text{-}\text{γ}}$, [4]

where the spectral shape function for b­_bp_ is expressed with γ calculated dynamically using a blue to green R_rs_ ratio following Lee et al. (2002). The amplitudes (M_x_) represent the desired unknowns (including b_bp_) and the Levenberg-Marquardt least squares method is used to determine those values that produce the lowest misfit between measured and reconstructed r_rs_.

**Text S2. Description of Sensitivity Results**

The basic idea of the sensitivity analysis is to hold the default GIOP configuration constant and allow one change to its parameterization. We chose parameterization changes for each of the spectral shape functions in GIOP (e.g., b_bp_ (**V1**)_,_ a_cdm_ (**V2**), a_ph_ (**V3**)) and we also ran GIOP without Raman-corrected R_rs_ (**V4**). We found that none of the choices in this GIOP sensitivity analysis produce a similar MPE to what is observed with CALIOP. However, spectral shape function choices do have an effect on the performance of MODIS b_bp_. The GSM b_bp_ power law exponent of -1 (with all other GIOP mechanics held constant) decreases the bias of MODIS b_bp_ beyond any of the other adjustments, although it does not significantly improve the MPE. Changing the a_cdm_ spectral shape function to that of the Quasi-Analytical-Algorithm (QAA, Lee et al., 2002) does not substantially change MODIS performance compared to the optimum, default configuration. Changing the a_ph_ spectral shape function from Bricaud et al. (1998) to Ciotti and Bricaud (2006) increases the bias by ~10% and results in a nearly 20% increase in MPE. Accounting for Raman scattering in this study did not improve the performance metrics of MODIS b_bp_.

| **V1** | **V2** | **V3** | **V4** |
| --- | --- | --- | --- |
| GSM b_bp_ shape  Maritorena et al., (2002) | QAA a_cdm_ shape  Lee et al., (2002) | Ciotti and Bricaud (2006) a_ph_ shape | R_rs_ without Raman correction |

**Table S1.** List of inversion variants used in Figure 3.

**Text S3. Description of CALIOP b_bp_ processing**

CALIOP’s cross-polarized channel is primarily due to backscatter from particles, but the parallel channel signal is contaminated by molecular scattering. In 2015, CALIOP tilted 30º off nadir twice per month to collect measurements with relatively low molecular backscatter so that this signal could be removed from the parallel channel (Lu et al., 2016). The off-nadir tilt allowed for the improvement of particulate depolarization ratios ($\delta_{p})$, and $\delta_{p}$ is compared with the diffuse attenuation coefficient (K_d,_ 532 nm) from airborne lidar campaigns rather than collocated MODIS data (where the mean $\delta_{p}$: K_d_ is 1.76, see Behrenfeld et al., 2017; 2019). Using these empirical linear relationships between depolarization and K_d_, particulate depolarization and K_d_ cancel out each other in the equation (as 1+ $\delta_{p}$ is very close to 1, and $\delta_{p}$ is roughly 2 K_d_).

Thus, the cross-polarization measurements of CALIPSO are directly proportional to the particulate backscatter, or

$$b_{bp}\left( 532 \right)=\frac{2K_{d} \beta_{w+}}{0.32* {0.98}^{2}} \frac{1+ \delta_{p}}{\delta_{p}} \approx\frac{2K_{d} \beta_{w+}}{0.32* {0.98}^{2}} \frac{1}{2K_{d}}\approx\frac{\beta_{w+}}{0.32* {0.98}^{2}}$$

CALIOP b_bp_ data were only used when cloud layers were <1 optical depth (defined by the detection limit for the remaining subsurface ocean signal). In addition, microwave scanning radiometer data (from AMSR-E/AMSR-2 at quarter degree resolution) were used to omit CALIOP retrievals at high wind speeds $\geq$ 9 m s^-1^ to avoid the influence of bubbles on b_bp_ (this eliminated ~10% of observations). Additionally, for lower winds, pixels that showed regionally anomalously high depolarization ratios were removed, as they are suspected to be contaminated by bubbles or sea ice. This removed an additional 3% of lidar data.

In our study we choose a beta(π)/b_bp_ value of 0.32. Because the Behrenfeld et al., (2019) CALIOP data were processed using a value of 0.16, we multiply the retrieved b_bp_ product (from http://orca.science.oregonstate.edu/lidar_grl_2020.php) by 0.5.

Text S4. Comparison of this study & Lacour et al., 2020

During the review of this manuscript, a regional comparison of MODIS, Argo, and CALIOP data in the North Atlantic was published (Lacour et al., 2020). While our focus is not to compare broad regional performance in these three sensors, it is worthwhile to discuss similarities and differences between the methods herein and those in Lacour et al. (2020).

In the Lacour et al. study, the only direct matchups between MODIS and CALIOP were performed on variable spatial scales. Spatially speaking, these are 9 km, 1^o^, and 2^o^ grids (where CALIOP and MODIS observations are binned). The time window separating a CALIOP observation from either a MODIS or Argo one was up to 16 days in all cases. CALIOP vs Argo, and MODIS vs Argo comparisons were not equivalent because the same Argo observations were not used to make the scatter plots.

The Lacour study finds superior performance of MODIS relative to CALIOP. This comparison may differ from our conclusions because the Argo observations used to make their comparisons were not common to both satellite sensors, except for when the cumulative distribution functions were compared across all platforms. Also, Argo K_d_ is used to compute CALIOP b_bp_ in the Lacour study, which may introduce differences in their CALIOP b_bp_ retrievals relative to ours, given that Argo and CALIOP observations may vary up to 16 days apart in a highly convective environment, especially for two sensors with small footprints. An additional reason for the relatively poor correspondence between CALIOP and Argo b_bp_ in the Lacour work is that CALIOP and MODIS are unbiased with respect to sampling, especially in environments with intermittent cloud cover and storms. A regional average from CALIOP is not necessarily equivalent to one from Argo.

Finally, the Lacour study uses a constant value (0.78) for the backscattering power-law exponent in order to calculate Argo values at 532 nm. In our study we use a variable spectral slope in space and time (as a function of R_rs_).

**Figure S1.** Probability histograms of MODIS and CALIOP b_bp_ by month, after the updated phase function value of 0.32 is used. Y-axis is normalized probability, and x-axis is b_bp_ (m^-1^).

**Figure S2.** Histograms of sampling month for +/-3 hours (left) and +/- 24 hours (right) across shared observations of MODIS, CALIOP, and Argo b_bp_.

**Figure S3.** A) CALIOP vs Argo b_bp_ (532 nm) for +/-3 hour matchups. B) MODIS vs Argo b_bp_ (532 nm) for +/-3 hour matchups. C) MODIS vs CALIOP b_bp_ (532 nm) for +/-3 hour matchups. D) Probability distributions for MODIS, CALIOP, Argo b_bp_ (532 nm) for +/-3 hours matchups. E) CALIOP vs Argo b_bp_ (532 nm) for +/-24 hour matchups. F) MODIS vs Argo b_bp_ (532 nm) for +/-24 hour matchups. G) MODIS vs CALIOP b_bp_ (532 nm) for +/-24 hour matchups. H) Probability distributions for MODIS, CALIOP, Argo b_bp_ (532 nm) for +/-3 hours matchups. The black line in A, B,C,E,F,G is the 1:1 line.

**Figure S4.** A) Bland-Altman plot between CALIOP and Argo where the x-axis is the means of paired (CALIOP, Argo) observations and the y axis is the percent error of CALIOP. The blue line is the mean bias and the red dashed lines are standard deviation of the error estimates. Points outside of the red lines can be viewed as outliers. B) Bland-Altman plot for MODIS and Argo b_bp_. C) Bland-Altman plot for MODIS and CALIOP b_bp_. D) Probability distributions for MODIS, CALIOP, Argo b_bp_. E) Bland-Altman plot for CALIOP and Argo b_bp_. F) Bland-Altman plot for MODIS and Argo b_bp_. G) Bland-Altman plot for MODIS and CALIOP b_bp_. H) Probability distributions for MODIS, CALIOP, Argo b_bp_ (532 nm). The top row is for +/-3 hour matchups and the bottom row is +/- 24 hour matchups. In all cases, b_bp_ is 532 nm.
